# High Fasting Plasma Glucose during Early Pregnancy: A Review about Early Gestational Diabetes Mellitus

**DOI:** 10.1155/2017/8921712

**Published:** 2017-10-18

**Authors:** E. Cosson, L. Carbillon, P. Valensi

**Affiliations:** ^1^Department of Endocrinology-Diabetology-Nutrition, AP-HP, Jean Verdier Hospital, Paris 13 University, Sorbonne Paris Cité, CRNH-IdF, CINFO, Bondy, France; ^2^Sorbonne Paris Cité, UMR U1153 Inserm/U1125 Inra/Cnam/Université Paris 13, Bobigny, France; ^3^Department of Gynecology-Obstetrics, AP-HP, Jean Verdier Hospital, Paris 13 University, Sorbonne Paris Cité, Bondy, France

## Abstract

Fasting plasma glucose (FPG) is nowadays routinely measured during early pregnancy to detect preexisting diabetes (FPG ≥ 7 mmol/L). This screening has concomitantly led to identify early intermediate hyperglycemia, defined as FPG in the 5.1 to 6.9 mmol/L range, also early gestational diabetes mellitus (eGDM). Early FPG has been associated with poor pregnancy outcomes, but the recommendation by the IADPSG to refer women with eGDM for immediate management is more pragmatic than evidence based. Although eGDM is characterized by insulin resistance and associated with classical risk factors for type 2 diabetes and incident diabetes after delivery, it is not necessarily associated with preexisting prediabetes. FPG ≥ 5.1 mmol/L in early pregnancy is actually poorly predictive of gestational diabetes mellitus diagnosed after 24 weeks of gestation. An alternative threshold should be determined but may vary according to ethnicity, gestational age, and body mass index. Finally, observational data suggest that early management of intermediate hyperglycemia may improve prognosis, through reduced gestational weight gain and potential early introduction of hypoglycemic agents. Considering all these issues, we suggest an algorithm for the management of eGDM based on early FPG levels that would be measured in case of risk factors. Nevertheless, interventional randomized trials are still missing.

## 1. Introduction

Gestational diabetes mellitus (GDM) was historically defined as “any degree of glucose intolerance with onset or first recognition during pregnancy,” whatever the treatment course and postpartum evolution [1]. Chronic insulin resistance in the second half of pregnancy is a central component of the pathophysiology of GDM [2, 3]. Diagnostic criteria were therefore established for 24–28 weeks of gestation (WG) including both glucose values during an oral glucose tolerance test (OGTT) and incident events [1]. The International Association of Diabetes Pregnancy Study Group (IADPSG) proposed the following diagnostic criteria: fasting plasma glucose (FPG) value ≥ 5.1 mmol/L and/or 1-hour glucose value ≥ 10.0 mmol/L and/or 2-hour glucose value ≥ 8.5 mmol/L. Although the National Institute for Health and Care Excellence (NICE) did not (http://www.nice.org.uk/guidance/ng3/evidence), these diagnostic criteria have been adopted worldwide, for example, by the American Diabetes Association (ADA) [4], the American Association of Clinical Endocrinologists [5], the Société Francophone du Diabète and the Collège National des Gynécologues et Obstétriciens Français [6], the International Federation of Gynecology and Obstetrics (FIGO) [7], and the Italian National Institute of Health (ISS) (http://www.salute.gov.it). The IADPSG criteria for the diagnosis of GDM are therefore now commonly called the 2013 WHO criteria for GDM (http://apps.who.int/iris/bitstream/10665/85975/1/WHO_NMH_MND_13.2_eng.pdf).

As the proportion of patients with unknown type 2 diabetes has been increasing, a new category of glucose disorder was introduced with the IADPSG recommendations. Women are considered to have overt diabetes [1] or diabetes in pregnancy (DIP (WHO criteria)) if their plasma glucose values are above the thresholds defining diabetes outside of pregnancy: FPG value ≥ 7 mmol/L and/or 2-hour glucose value ≥ 11.1 mmol/L and/or HbA1c ≥ 6.5%. Preconceptional diagnosis of diabetes or prediabetes would actually be useful to optimize glucose levels prior to conception but is rarely performed. On another hand, waiting for 24 WG to diagnose DIP would delay care in an unacceptable way and potentially lead to severe obstetric complications, as obstetric outcomes are quite similar for undiagnosed type 2 and type 1 diabetes [8]. Furthermore, the prevalence of fetal malformations was recently reported to be higher in women with DIP compared to women with GDM [9]. The incidence of other outcomes was similar in both groups [9].

Concomitantly, the IADPSG recommended using a FPG range 5.1–6.9 mmol/L before 24 WG to define early GDM (eGDM). Measurement was recommended at the first prenatal visit, or later if undone, especially in high-risk women [1]. The threshold of 5.1 mmol/L is arbitrary and was chosen as the same value as after 24 WG. Actually, FPG was described to be quite stable during pregnancy [10]. In this study, FPG levels were similar in the same women when measured at 17 WG then at 32 WG [10], despite an increase in insulin secretion and a propensity for earlier hypoglycemia during fasting as described a long time ago [11]. The IADPSG recommends that women with eGDM should be referred for immediate care, even if the level of proof for this recommendation is very low regarding to prognosis [12, 13]. Alternatively, some guidelines do not recommend to treat immediately women with high FPG levels in early pregnancy, such as the ADA [4]. Other advice for patients who have an early FPG between 5.6 and 6.9 mmol/L is to perform an OGTT at 16–18 WG and to monitor and treat abnormal results at this time (http://www.salute.gov.it).

## 2. Methods

References for this review were identified through searches of PubMed for articles published until March 2017, by use of the terms “early gestational diabetes mellitus” and “fasting plasma glucose and pregnancy.” English and French articles resulting from these searches and relevant references cited in those articles were reviewed. We discuss here the prevalence, risk factors, and metabolic characteristics of eGDM, as well as its prognosis.

## 3. Results

### 3.1. Bibliography

Our search in Medline suggested 1800 references between January 2010 and March 2017. Of these articles, we selected and analysed around 100 articles. We additionally analysed around 20 articles that were cited in these papers. We also found 5 relevant ongoing studies in clinicaltrials.gov. We finally selected 49 references.

### 3.2. The Burden of eGDM

#### 3.2.1. Diagnosis with Early FPG Measurement

A FPG level ≥ 5.0 mmol/L was reported in 11.9% of pregnant women during the first trimester of pregnancy (mean 9 WG) in Israel [14], where universal screening for FPG has been recommended at the first prenatal care visit. This prevalence was close to the prevalence of FPG above 5.1 mmol/L that was reported at the first prenatal visit in a multicenter study in China [15] and in one center in Italy [16] (11.4% and 7.2%, resp.), noting that almost all women were tested in these studies.

In France, where selective screening and using IADPSG criteria is recommended, data from the French National Interscheme Health Insurance Information System (SNIIRAM) showed that only 2.3% of 788,494 pregnant women in 2013 without known diabetes were treated for a dysglycemia diagnosed before 22 WG [17]. These women corresponded to 26.9% of all women with dysglycemia during their pregnancy. The French criteria for selective screening are maternal age ≥ 35 years, body mass index (BMI) ≥ 25 kg/m^2^, history of diabetes in a first-degree relative, personal history of GDM, or having giving birth to a child with fetal macrosomia [6]. In the United States, implementing early screening nearly doubled the incidence of GDM as compared with a previous standard two-step approach [18].

#### 3.2.2. Diagnosis with Early OGTT

A high prevalence of eGDM has also been reported when OGTT is performed in early pregnancy in selected populations with risk factors. The prevalence was 23.4% in pregnant women with a BMI at or above 29.0 kg/m^2^ in early pregnancy as part of the enrollment into the DALI (Vitamin D And Lifestyle Intervention for GDM prevention) pilot and lifestyle Pan-European multicenter trials [19]. Additionally, the prevalence of eGDM in high-risk populations was recently reported to be 48.8% in Italy [20] and 27.3% in Australia [21].

To conclude this part, intermediate FPG levels in early pregnancy, also known as eGDM, have become a very common issue.

### 3.3. Risk Factors for Hyperglycemia in Early Pregnancy

**Figure 1:**
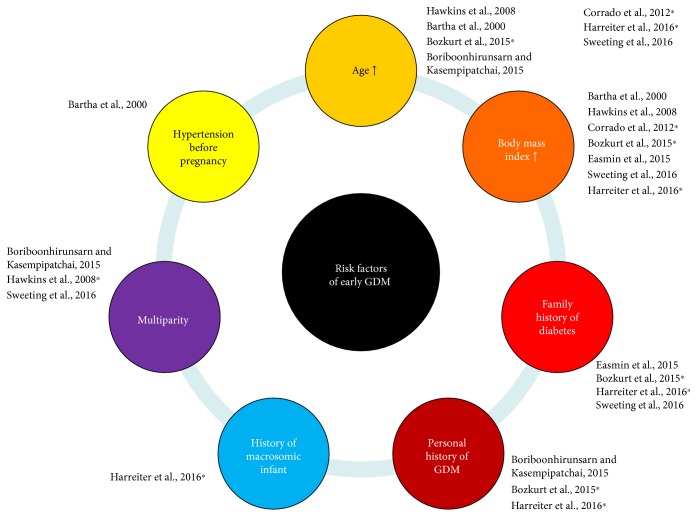
Risk factors for early gestational diabetes mellitus. GDM: gestational diabetes mellitus. ∗ identifies references where early GDM is defined according to the IADPSG definition, that is, fasting plasma glucose value ≥ 5.1 mmol/L.

Figure 1 shows that FPG ≥ 5.1 mmol/L during early pregnancy is more prevalent with aging [16, 19, 22], higher BMI [16, 19, 22], a family history of diabetes [19, 22], a personal history of GDM [19, 22] or newborn with macrosomia [19], and multiparity [23].

Studies using other definition for eGDM found similar risk factors [21, 23–26]. However, all those results should be interpreted with caution as screening is usually performed in already high-risk subjects.

### 3.4. Metabolic Characteristics of Women with eGDM

Bozkurt et al. have evaluated pathophysiological characteristics of pregnant women diagnosed with GDM according to IADPSG criteria [22]. Unlike patients with late (regular) GDM or normal glucose tolerant women, subjects with eGDM exhibited decreased insulin sensitivity, with lower oral glucose insulin sensitivity index and quantitative insulin sensitivity check index. The subgroups also differed in BMI, with significantly higher levels in patients with eGDM compared to subjects with late GDM and those with normal glucose tolerance (31.7 ± 6.4, 27.7 ± 4.4, and 27.3 ± 5.6 kg/m^2^, resp., *p* < 0.001). However, differences in estimated insulin sensitivity remained significant after adjustment for BMI, age, and history of GDM. In this study, subjects with either early or late GDM showed impairments in *β*-cell function (insulinogenic index) as compared with women without GDM [22].

In another study including women with BMI at or above 29 kg/m^2^ who were screened in early pregnancy using a 75 g OGTT, various indexes of insulin sensitivity (oral glucose insulin sensitivity index, quantitative insulin sensitivity check index, and homeostasis model assessment of insulin resistance (HOMA-IR)) and secretion (Stumvoll first and second phases) decreased progressively in women with normal glucose tolerance, eGDM, and DIP [19]. Differences persisted after adjustment for age, pregestational BMI, gestational week, and fetal gender [19].

### 3.5. What Does eGDM Outside Pregnancy Mean?

**Figure 2:**
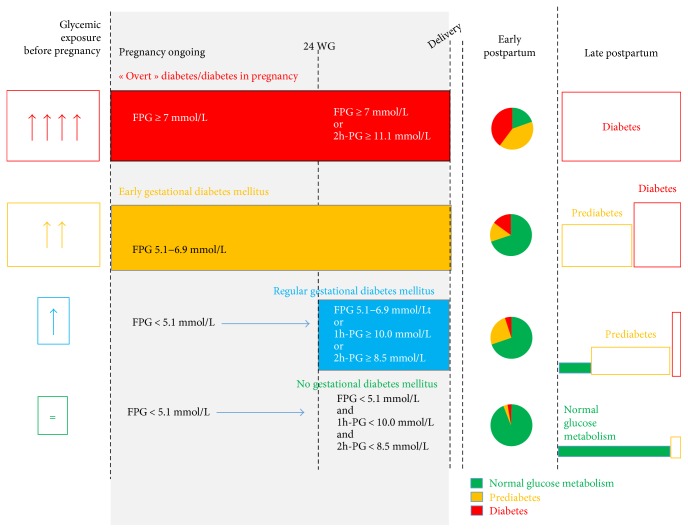
Does eGDM mean preexisting prediabetes? The hypothesis is when hyperglycemia has been present (but unknown) before pregnancy, then fasting plasma glucose (FPG) is already increased during early pregnancy while insulin resistance increases after 24 weeks of gestation (WG). Accordingly, oral glucose tolerance will reveal dysglycemia in early postpartum. 1h-PG and 2h-PG: plasma glucose 1 and 2 hours after 75 g oral glucose tolerance test; FPG: fasting plasma glucose; WG: weeks of gestation.

Mills et al. [27] have shown that there is a physiological reduction in FPG concentration in normal pregnancy. In their cohort of 361 healthy pregnant women, they showed that FPG levels decrease with advancing pregnancy with a plateau occurring around 10–20 WG [27]. Thereafter, insulin resistance increases and “late” GDM may occur [28]. As shown and explained in Figure 2 and as it was previously suggested [29], high FPG during early pregnancy could indicate unknown prediabetes (if eGDM) or diabetes (if DIP) before pregnancy as insulin resistance remains present in both cases. Although glycemic status before pregnancy is usually unknown, we may use several indicators, such as markers for glycemic exposure before pregnancy, early postdelivery glycemic status (which is usually considered to reflect glycemic status before pregnancy), and incidence of type 2 diabetes postpartum.

#### 3.5.1. Marker for Long-Term Glycemic Exposure at the Time of eGDM Diagnosis

Skin autofluorescence, a measurement of cutaneous advanced glycation end products, can be used as a screening method in detecting unknown diabetes. Maury et al. have suggested that skin autofluorescence was a marker of metabolic memory in pregnant women. During pregnancy, forearm skin autofluorescence at 24–30 WG was reported to gradually decrease from patients with previous diabetes, to women with GDM and previous hyperglycemia, to those with GDM without previous hyperglycemia, and finally to normal subjects without diabetes or GDM [30]. In that study, previous hyperglycemia was defined as previous GDM or having given birth to a macrosomic infant or GDM diagnosed before 24 WG (eGDM) [30].

#### 3.5.2. Data Based on OGTT in the Immediate Postpartum

Sweeting et al. have recently reported the results of postpartum OGTT according to the time of GDM diagnosis [21]. In this study, women were tested between 2001 and 2011, with early screening performed in women considered at high risk for GDM. The repartition of normal plasma glucose values, impaired glucose tolerance, and diabetes during OGTT performed three months postpartum significantly differed according to eGDM < 12 WG (normal glucose tolerance 79%, impaired glucose tolerance 11%, and diabetes 11%), GDM 12–23 WG (71, 24, and 5%, resp.), and GDM > 24 WG (85, 14, and 1%, resp.). However, the proportion of dysglycemia was only 22% in women with eGDM < 12 WG, suggesting that eGDM is not a good marker of preexisting dysglycemia. However, the implementation of a lifestyle change program during pregnancy and postpartum might partially account for this result.

#### 3.5.3. Postpartum Development of Type 2 Diabetes

The presence of eGDM could also identify women with an increased risk to later develop type 2 diabetes. In a systematic review including 8 studies and 4026 women with GDM defined with numerous criteria, women with eGDM had a twofold increased risk of incident type 2 diabetes 6 weeks to 20 years after delivery compared to subjects with “late” GDM (relative risk (RR) 2.13 (95% CI 1.52–3.56)) [31]. IADPSG-diagnosed eGDM has also been reported to be a significant predictor of progression to abnormal glucose tolerance up to 5 years postpartum. In this study, the earlier the GDM was diagnosed, the higher was the rate of dysglycemia up to 5 years postpartum [32].

To conclude at this step, although eGDM is associated with a profile comparable to metabolic syndrome, with higher insulin resistance than in “regular GDM,” it has not been consistently associated with prediabetes or diabetes during early postpartum. However, it is associated with more incident type 2 diabetes than regular GDM (Figure 2). Overall, this suggests that eGDM should be considered as a new intermediate entity between normal glucose metabolism and prediabetes/diabetes outside pregnancy.

### 3.6. The Meaning of an Early High FPG through Pregnancy

#### 3.6.1. Persistence through Pregnancy

eGDM was initially supposed to be persistent through pregnancy. Therefore, it was hypothesized that early care for eGDM would improve prognosis and that was the reason why FPG in the range 5.1–6.9 mmol/L was recommended to diagnose GDM at any time during pregnancy [1]. However, data from Italy [16] and China [15] have challenged this recommendation. Actually, at least 50% of the women with eGDM have no GDM after 24 WG, despite the absence of specific care. In the Italian publication, 55% of women with FPG ≥ 5.1 mmol/L during early pregnancy had a normal OGTT after 24 WG [16]; in the Chinese study, less than one-third of the women still had a FPG ≥ 5.1 mmol/L between the first prenatal visit and 24–28 WG [15].

#### 3.6.2. Does Early FPG Predict GDM after 24 WG?

Smirnakis et al. showed in a prospective study that women in whom GDM was diagnosed at 24–28 WG (using the O'Sullivan and Mahan criteria of the American Diabetes Association) demonstrated higher levels of FPG (4.8 ± 0.6 versus 4.4 ± 0.4 mmol/L, *p* < 0.05) and HOMA-IR at 17 WG compared to women who had normoglycemic pregnancies [33]. Riskin-Mashia et al. have also shown that first-trimester FPG levels were positively associated with the risk of GDM after 24 WG, especially when values were ≥5.0 mmol/L [14]. In another study including overweight women at very high risk for GDM, the prevalence of IADPSG-defined GDM after 24 WG was 53% in those with early FPG ≥ 4.9 mmol/L whereas the prevalences were 15, 12, and 20% in those with early FPG ≤ 4.4, 4.41–4.6, and 4.61–4.89 mmol/L, respectively [34]. Therefore, FPG in early pregnancy can be considered as a tool to select the women at risk for GDM after 24 WG. For example, the sensitivity, specificity, positive, and negative predictive values of FPG > 4.4 mmol/L in early pregnancy to predict IADPSG-defined GDM in an Italian population were 80%, 66%, 77%, and 96%, respectively [35]. Therefore, using the recursive portioning and amalgamation method, it was suggested to consider a low FPG to avoid OGTT after 24 WG because of its high negative predictive value [35]. Actually, the negative predictive value was not so high in two other studies. First, in a Chinese population, the sensitivity, specificity, positive, and negative predictive values of FPG > 4.4 mmol/L to predict IADPSG-defined GDM were 78%, 38%, 21%, and 89%, respectively [15]. Second, in overweight women at very high risk of GDM, the negative predictive value of early FPG ≤ 4.4 mmol/L was only 85% [34].

Additionally, FPG concentration at first antenatal visit in 2284 women in China was higher in those who later developed GDM than in those who did not. However, early FPG was associated with late GDM when measured between 12 and 16 WG, 16–20 WG and 20–24 WG, but not before 12 WG. This suggested that the relationship between FPG and late GDM appeared during the second trimester [36].

### 3.7. Should We Consider Alternatives to FPG Measurement in Early Pregnancy

#### 3.7.1. The Limits of Measuring FPG in Early Pregnancy

Analyzing FPG level is actually complex. First, FPG decreases with increasing gestational age [15]. For example, in a Chinese population [15], median FPG was 4.95 mmol/L at 4–6 WG, 4.70 at 10–12 WG, and 4.53 at 14–16 WG and was the lowest at 4.38 mmol/L at 20–24 WG.

Second, based on the receiving operating curves and area under the curve measurements, in another Chinese population, the optimal FPG cut-off values to predict GDM after 24 WG were different according to BMI categorization groups [37]. The FPG cut-off value was 4.77 mmol/L in prepregnancy underweight women, 4.92 mmol/L in prepregnancy normal weight women, 5.00 mmol/L in prepregnancy overweight women, and 5.05 mmol/L in prepregnancy obese women [37].

Overall, the threshold for FPG could be different according to WG and to BMI level. There is a need to investigate the best threshold for early FPG and the association with late GDM or poor pregnancy outcomes. The concordances of early OGTT, at 12–15 WG [38] or at 18–20 WG [39], are going to be compared to OGTT results at 24–28 WG in two studies, but no data are currently available yet.

#### 3.7.2. OGTT

OGTT could be more sensitive than FPG alone to diagnose GDM during early pregnancy. For example, in pregnant women with obesity at 15.2 ± 3.0 WG and using IADPSG criteria, 1- and 2-hour glucose values led to diagnose 21.5% additional GDM as compared to FPG value during the OGTT [19]. However, data on 1- and 2-hour OGTT glucose values are rare in early pregnancy. Using IADPSG criteria is not evidence based in early pregnancy. Especially, thresholds that are used after 24 WG are too high for early pregnancy [10]. OGTT is time-consuming, inconvenient, and uncomfortable, inducing nausea and vomiting in some patients. Therefore, some women might refuse to repeat OGTT after 24 GW.

#### 3.7.3. HbA1c Measurement

HbA1c measurement also warrants further evaluation [13]. HbA1c ≥ 5.9% has been reported to identify all cases of DIP and to be associated with poor pregnancy outcomes, including congenital anomalies, preeclampsia, shoulder dystocia, and perinatal deaths [40]. The association with poor outcomes appears to be independent of later GDM diagnosis [41]. However, HbA1c may vary with pregnancy hemodilution [42] and with the presence of hemoglobinopathy and/or anemia.

To conclude this part, FPG > 5.1 mmol/L persists in less than one-half of untreated women and we should define an alternative FPG threshold to define eGDM. However, it may differ according to BMI and WG.

### 3.8. Prognosis Related to Hyperglycemia in Early Pregnancy (Table 1)

#### 3.8.1. Poor Pregnancy Prognosis Related to FPG in Early Pregnancy

A poor prognosis of high FPG levels during the first trimester was reported, with an increased risk of adverse pregnancy outcomes, including macrosomia [14, 36] and cesarean section [14].

Among the 788,494 women who delivered in France in 2013, women with early onset of GDM were more likely to have need for cesarean section (odds ratio 1.10 (95% confidence interval 1.05–1.15)) and large for gestational age infants (1.18 (1.12–1.24)) than women diagnosed between 22 and 30 WG [17].

#### 3.8.2. Poor Prognosis Associated with eGDM Not Defined with FPG

Bartha et al. [24] have compared complications associated with GDM diagnosed in early (mainly during the first trimester) or late pregnancy. eGDM screening was performed only in case of risk factors, especially increased BMI, which was a bias as the presence of risk factors has been associated with a worse prognosis [43]. They reported that the group diagnosed earlier in pregnancy had higher rates of preeclampsia, neonatal hypoglycemia, and perinatal deaths and lower rate of oligohydramnios [24]. Preeclampsia, shoulder dystocia, macrosomia, and hyperbilirubinemia were reported to be more frequent in women with diet-treated GDM diagnosed before than after 24 WG, even after adjustment for maternal age, ethnicity, parity, weight, and blood glucose control [23]. Obstetric outcomes were also compared in early onset and late onset GDM in Bangladesh and showed a poorer prognosis associated with eGDM, including more preeclampsia, neonatal admission in intensive care unit, and neonatal hypoglycemia [26].

When using the Australasian Diabetes in Pregnancy Society diagnostic criteria in a large Australian multiethnic cohort of women considered at high risk for GDM, eGDM was also associated with poorer pregnancy outcomes [21]. In this study, the eGDM cohort was a preselected high-risk group and patients with eGDM diagnosed before 12 WG had an intermediate risk profile, standing between the risk of subjects with preexisting type 2 diabetes and the risk of patients diagnosed with eGDM between 12 and 23 WG. The outcomes in women who were diagnosed with GDM before 12 WG were quite comparable to those observed in subjects with preexisting diabetes despite early testing and current best practice treatment [21].

Further research is currently necessary to evaluate the effects of early metabolic changes on short- and long-term outcomes for the mother and the child and of the potential consequences on generational transmission of metabolic diseases.

### 3.9. Does the Treatment of Hyperglycemia in Early Pregnancy Improve Prognosis? (Table 1)

#### 3.9.1. Argumentation from Retrospective Studies Comparing Women Cared for Early or Regular GDM

Early detection and treatment of women at high risk of eGDM might improve pregnancy outcomes: several studies have shown a similar prognosis in early and late GDM with treatment. For example, an Indian team assessed the merits of care given to women in whom GDM was diagnosed in different WG in order to find out the ideal period of screening in women with history of high-risk pregnancies. The babies born to GDM women diagnosed before 12 WG had a lower birth weight than the ones born to GDM women diagnosed after 30 WG. The authors concluded therefore that screening in the first trimester of pregnancy and institution of therapy was advisable in women with high-risk pregnancies [44]. In Thailand, the incidence of pregnancy complications was similar in women diagnosed with GDM early and late in pregnancy [25]. In this study, gestational weight gain and glycemic control, but not the time of diagnosis, were independently associated with macrosomia in women with GDM [25]. The authors suggested that the higher frequency of insulin treatment, as consistently reported [17, 18, 21, 24–26, 45], and the lower gestational weight gain [21, 24, 25] in women with eGDM compared to women with late GDM might explain a similar prognosis in both groups in these studies. However, although insulin initiation was also earlier in eGDM than in late GDM, with a higher maximum daily insulin dose in the study by Sweeting et al., the prognosis was worse in women with eGDM [46].

#### 3.9.2. Argumentation from Retrospective Studies Comparing Strategies including Early Screening for Dysglycemia or Not

Alunni et al. compared two cohorts of pregnant women with GDM: those diagnosed via two-step screening (standard approach) versus those diagnosed via early screening diagnosis with additional screening after 24 WG if early screening was negative. The second approach doubled the incidence of GDM, but there was no significant difference in neonatal outcomes [18].

Hong et al. analyzed a retrospective cohort of women with singleton pregnancies diagnosed with GDM who had indications for early screening, defined as the presence of obesity, or GDM or macrosomia in a prior pregnancy. Women were classified as having been screened early (<20 WG) or routinely (>24 WG). The decision of whether or not a patient had early screening for GDM was at the discretion of the managing provider. Early screening was not associated with significant reduction in the risk of cesarean, preeclampsia, macrosomia, or birth injury. The authors concluded that the utility of early GDM screening still required an evaluation [45].

#### 3.9.3. Future Randomized Trials

Randomized controlled trials evaluating the benefit-cost balance for screening and treating less severe hyperglycemia than DIP in early pregnancy are mandatory [13]. We have found several ongoing trials. One will compare the prognosis of pregnancies of 202 women with first trimester hyperglycemia (defined with FPG or HbA1c) when treatment begins before 15 WG versus after 28 WG (clinicaltrials.gov NCT01926457). The Early Gestational Diabetes Screening in the Gravid Obese Woman (EGGO, clinicaltrials.gov NCT01864564) and the Randomization of Early Diabetes Screening Among Obese Pregnant Women (REDSOAP, clinicaltrials.gov NCT03116009) studies will include 1150 and 600 obese women, respectively. They will compare the prognosis associated with screening strategies including or not a screening for dysglycemia during early pregnancy. Finally, the Early Diagnosis of Gestational Diabetes Mellitus Study (EDoGDM) will randomize 600 low-risk pregnant women with an OGTT performed at 18 to 20 WG versus 24 and 28 WG and will compare prognosis in both groups ([39], clinicaltrials.gov NCT02740283).

To conclude this part, high FPG during early pregnancy is associated with a poor prognosis, which might be improved with immediate care through diet and insulin therapy if necessary. However, the results of randomized trials are still lacking.

### 3.10. What Could Be Proposed in Clinical Practice at the Current Time?

**Figure 3:**
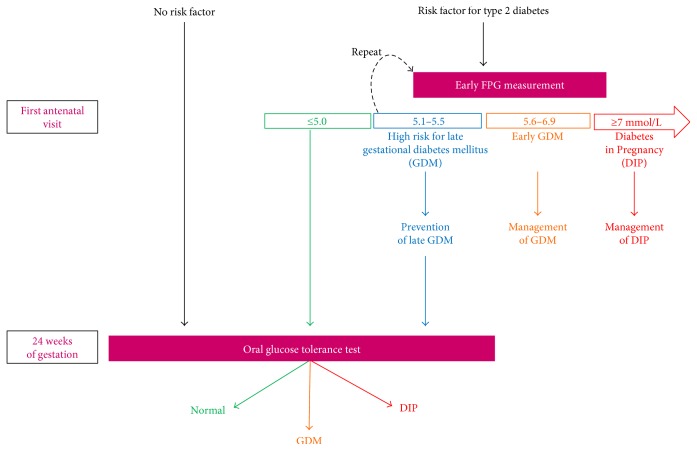
Proposals for a management algorithm according to the presence of risk factors and screening for dysglycemia during pregnancy. DIP: diabetes in pregnancy; FPG: fasting plasma glucose; GDM: gestational diabetes mellitus.

We propose the following algorithm of management according to early FPG (Figure 3). This proposal has not been validated by an expert consensus and should obviously not be considered as validated recommendations. The increasing number of subjects with undiagnosed type 2 diabetes mellitus before pregnancy justifies the screening of women with risk factors for preexisting diabetes by the first antenatal visit. We currently suggest to keep diagnosing DIP when FPG is 7.0 mmol/L or above. Actually, the incidence of macrosomia, preeclampsia, and neonatal hypoglycemia has been reported to be similar in treated women with DIP or GDM [9]. As eGDM is also associated with classical risk factors for type 2 diabetes, we suggest, for feasibility reasons, to screen early in pregnancy only the subjects considered to be at high risk. We suggest to test subjects with characteristics predisposing to type 2 diabetes according to the local frequency of abnormal glucose metabolism and according to local guidelines.

The current recommended FPG threshold (5.1 mmol/L) to define eGDM is an issue, as FPG depends on gestational age, ethnicity, and BMI categories [13, 15, 37]. We suggest to refer for care any subject with a FPG between 5.6 and 6.9 mmol/L as the risk of giving birth to a child with macrosomia increases with higher FPG [14, 36, 47]. We acknowledge that a FPG threshold at 5.6 mmol/L is also arbitrary as the association between early FPG and pregnancy outcomes appears to be a continuum [48]. However, Zhu et al. [15] showed that the positive predictive value of an abnormal OGTT at 24–28 weeks was 60% from an early FPG 5.7 mmol/L, and last but not the least, Riskin-Mashiah et al. [14] reported in 6129 women with early FPG that the risk of macrosomia and primary cesarean section almost tripled and doubled, respectively, from this same threshold. Accordingly and as shown in the FIGO report [7], FPG values between 5.6 and 6.9 mmol/L are considered as GDM in China, Latin America, and the UK.

Because women might have inadequately fasted before the first measurement and because the reproducibility of FPG is imperfect, we propose to recheck the level of FPG when it is between 5.1 and 5.5 mmol/L and to consider the lowest value among both FPG measurements. HbA1c measurement might be an alternative to the second FPG but warrants further evaluation [13]. If the level of FPG is confirmed to be between 5.1 and 5.5 mmol/L, we suggest lifestyle changes (nutrition and exercise). Actually, the prevention of GDM seems more effective when started before 15 WG [49]. These women should be screened again, with OGTT, after 24 WG.

We think that all the remaining women, even those with a low early FPG level, should be screened after 24 WG. Actually, an early FPG < 4.4 mmol/L had a very high negative predictive value for late GDM in one study [35] but this was not confirmed in two other studies [15, 34]. Therefore, we do propose to keep a late screening in these women. Noteworthily, the screening after 24 WG includes women without risk factor but we have previously reported that women with GDM, even without risk factor and with adequate management, have a poor prognosis [43].

## 4. Conclusions

Measuring FPG in early pregnancy appears to be crucial to diagnose DIP and treat this condition as early as possible. Intermediate FPG levels in early pregnancy, also known as eGDM, have become a frequent issue. eGDM appears to represent a new intermediate entity in the following continuum: normal glucose metabolism, late GDM, eGDM, DIP, and pregravidic diabetes. Indeed, women diagnosed with eGDM have more risk factors for prevalent type 2 diabetes compared to subjects diagnosed with GDM later in pregnancy. They also have more incident type 2 diabetes and are more insulin resistant. This might participate to a poorer prognosis. However, there are major remaining issues about this new entity. Indeed, (i) a FPG > 5.1 mmol/L has been reported to persist in less than one-half of untreated women and the definition of eGDM needs to be better determined and (ii) there is nowadays no clear evidence of the usefulness of its treatment.

## Figures and Tables

**Table 1:** Data considering prognosis of early fasting plasma glucose, of early gestational diabetes mellitus, and of strategies including early screening for gestational diabetes mellitus or not.

Reference	*n*	Population	Screening methods for glycemic disorders	Predictive factors of eGDM	Care and differences during pregnancy (eGDM versus remaining)	Prognosis of eGDM (^∗^adjusted)
*Fasting plasma glycemia & prognosis*						
Riskin-Mashiah et al., 2009 [14]	6129 women	Retrospective Israel	FPG measurement at 9.5 (7.6–11.6) GW classified in 7 HAPO categories	NA	NA	Late GDM development ↑^∗^ LGA/macrosomia ↑^∗^ Cesarean delivery ↑^∗^
Liu et al., 2014 [36]	2284 women	Retrospective China	FPG at first antenatal visit (17.4 ± 4.6 GW)	NA	NA	Late GDM development ↑^∗^ Neonatal birth weight ↑^∗^
*eGDM & prognosis*						
Bartha et al., 2000 [24]	50 eGDM versus 133 regular GDM (24–28 WG)	Retrospective Case control Spain	Women with risk factors O'Sullivan then 100 g OGTT	Age ↑ BMI ↑ Hypertension ↑	GWG ↓ Insulin therapy ↑	Total preeclampsia ↑ Oligohydramnios ↓ Neonatal hypoglycemia ↑ Perinatal death ↑ Prematurity, fetal anomalies, cesarean section, SGA, macrosomia, NICU =
Hawkins et al., 2008 [23]	339 eGDM (<24 WG) versus 2257 regular GDM	Retrospective Case control United States	Diet-treated GDM Early screening in women with risk factors O'Sullivan then 100 g OGTT	Age ↑ BMI ↑ Multiparity ↑	DIET ONLY (insulin-treated women were excluded) Better decrease in fasting plasma value during follow up for eGDM group	Preeclampsia ↑^∗^ Shoulder dystocia ↑ Macrosomia ↑ Hyperbilirubinemia ↑^∗^ Shoulder dystocia, cesarean section, NICU, neonatal death =
Seshiah et al., 2008 [44]	120 NGT versus 87 GDM < 12 WG versus 18 GDM 13–23 WG versus 15 GDM 24–30 WG versus 18 GDM > 30 WG	Retrospective Case control India	Women with family history of diabetes and bad obstetric history 75 g OGGT: 2 h PG > 140 mg/dL	FPG, 2 h PG, and HbA1c are the highest in women with GDM < 12 GW	Unknown	Birth weight GDM < 12 GW lower than birth weight GDM > 30 GW
Easmin et al., 2015 [26]	60 eGDM (<24 WG) versus 60 regular GDM (24–32 WG)	Prospective observational Case control Bangladesh	Unknown	BMI ↑ Family history of diabetes ↑	Insulin therapy ↑	Preeclampsia ↑ NICU ↑ Neonatal hypoglycemia ↑ Hyperbilirubinemia, asphyxia, perinatal death =
Boriboonhirunsarn and Kasempipatchai, 2015 [25]	142 women with eGDM (<20 GW) versus 120 women with regular GDM	Thailand 2014	Women with risk factors O'Sullivan then 100 g OGTT in early and late pregnancies	Age ↑ Previous history of GDM ↑ Multiparity ↑	GWG↓ Insulin therapy↑ Better glycemic control	Term, preeclampsia, cesarean delivery, macrosomia, hyperbilirubinemia =
Sweeting et al., 2016 [21]	3493 GDM ≥ 24 GW versus 1247 GDM 12–23 GW versus 68 GDM < 12 GW versus 65 preexisting diabetes	Retrospective Case control	Early screening only in women with risk factors ADIPS criteria	Age ↑ BMI ↑ Family history of diabetes ↑ Multiparity ↑	GWG↓ Insulin therapy↑ Earlier insulin therapy↑ Maximum daily insulin dose↑	Gradient in 4 groups Preterm delivery ↑ Cesarean section ↑ Hypertensive disorders ↑ Macrosomia ↑ Hyperbilirubinemia ↑ Respiratory distress syndrome ↑
Regnault et al., 2016 [17]	18,299 women with eGDM (<22 GW) versus 37,551 women with GDM (22–30 GW)	From the 788,494 women who delivered in France in 2013	Early and regular screening in case of risk factors IADPSG criteria	No data	Insulin therapy ↑	Cesarean delivery ↑ LGA ↑
*Screening strategy including early screening & prognosis*						
Alunni et al., 2015 [18]	First period: 147 women with GDM after 24 GW versus second period: 175 GDM women with early and, if negative screening, late screening	From 2652 women who delivered between 2010 and 2012 United States	First period: O'Sullivan and 100 g OGTT after 24 GW Second period: early screening FPG and HbA1c; 75 g OGTT (IADPSG)	BMI ↓ during the second period	Comparison between first and second periods Insulin = Comparison during the second period (eGDM versus regular GDM) Insulin therapy ↑	Term = Cesarean delivery = Macrosomia = SGA =
Hong et al., 2016 [45]	112 women screened early (including 85 with early GDM and 27 with regular GDM) versus 457 women with regular GDM who were not tested earlier	569 women with risk factors for GDM United States	Early screening only in case of risk factors In early and regular screening: O'Sullivan then 100 g OGTT	Early screening strategy Private insurance ↑ History of GDM ↑ Chronic hypertension ↑ BMI ↑	Insulin therapy ↑	Early screening strategy Cesarean section =^∗^ Preeclampsia =^∗^ SGA and LGA =^∗^ Birth injury =^∗^ Preterm delivery ↑^∗^

2h-PG: 2-hour plasma glucose; ADIPS: Australasian Diabetes in Pregnancy Society; BMI: body mass index; eGDM: early gestational diabetes mellitus; GDM: gestational diabetes mellitus; GWG: gestational weight gain; HAPO: hyperglycemia and adverse pregnancy outcome; LGA: large for gestational age; NA: nonapplicable; NICU: neonatal intensive care unit; OGTT: oral glucose tolerance test; SGA: small for gestational age; WG: weeks of gestation; =: similar; ↓: decreased or lower; ↑: increased or higher. ^∗^References where multivariate analyses were performed.

## Abbreviations

ADA: American Diabetes Association
BMI: Body mass index
DIP: Diabetes in pregnancy
eGDM: Early gestational diabetes mellitus
FIGO: International Federation of Gynecology and Obstetrics
FPG: Fasting plasma glucose
GDM: Gestational diabetes mellitus
HOMA-IR: Homeostasis model assessment of insulin resistance
IADPSG: International Association of Diabetes Pregnancy Study Group
NICE: National Institute for Health and Care Excellence
OGTT: Oral glucose tolerance test
WG: Weeks of gestation.
